# Pre-Pectoral One-Stage Breast Reconstruction with Anterior Coverage Using Superior Anterior Biological Acellular Dermal Matrix (ADM) and Inferior Anterior Dermal Sling Support

**DOI:** 10.3390/medicina58080992

**Published:** 2022-07-25

**Authors:** Andrea Sisti, Payam Sadeghi, Roberto Cuomo, Sonia M. Alvarez

**Affiliations:** 1 Division of Plastic Surgery, University of Tennessee Health Science Center, Memphis, TN 38163, USA; salvarez@uthsc.edu; 2 Plastic Surgery Department, Cleveland Clinic, Cleveland, OH 44195, USA; drpayamsadeghi@yahoo.com; 3 Plastic and Reconstructive Surgery Division, Department of Medicine, Surgery and Neuroscience, “Santa Maria alle Scotte” Hospital, University of Siena, 53100 Siena, Italy; robertocuomo@outlook.com

**Keywords:** acellular dermal matrix, prepectoral breast reconstruction, ADM, implant, inverted-T mastectomy, skin-reducing mastectomy, immediate implant reconstruction, one-stage breast reconstruction

## Abstract

The use of acellular dermal matrix (ADM) implants has enhanced breast reconstruction. ADM is a biotechnologically designed human tissue of bovine or porcine origin in which tissue processing removes cellular antigens. In this case report, we describe the use of ADM in one-stage prepectoral breast reconstruction. Skin-reduction breast reconstruction with a prepectoral implant was performed. We created a combined dermal pocket using the inferior dermal flap, sutured with a patch of acellular dermal matrix to continue its extension until the upper pole, to cover the implant. This technique offers single-stage immediate reconstruction, with a decreased requirement for ADM and increased use of vascularized tissue and implant support. Additionally, in the pre-pectoral space, decreased pain postoperatively and less anatomic disruption is offered.

## 1. Introduction

Breast cancer is one of the most common cancers, with an increasing incidence in the female population [1]. Perhaps improvements in early detection and modern techniques of primary prevention contribute to this data. In conjunction with treatment for breast cancer, breast reconstruction and options for such are standard of care. Numerous techniques for breast reconstruction exist, including autologous tissue reconstruction, implant-based reconstruction or a combination of techniques, depending on the indication [2].

Implant-based breast reconstruction is the most frequently performed type of reconstruction [1]. Advancements in implant-based reconstruction techniques have resulted in higher aesthetic satisfaction as well as demand. Over the years, these new techniques have been proposed, giving life to a new surgical discipline called ‘oncoplasty’ [3,4,5]. Additionally, patients desire fewer steps, less disruption of anatomy and more comfort with reconstructive techniques. Pre-pectoral reconstruction as well as direct-to-implant reconstruction have become norms. 

The use of acellular dermal matrix (ADM) implants has enhanced breast reconstruction and other plastic surgery techniques [6].

ADM application in prepectoral breast reconstruction has already been described [7,8]. Skin-reduction breast reconstructions with a prepectoral implant was firstly described by Caputo et al. [9] in 2016. They created a combined dermal pocket using the inferior dermal flap, sutured with a patch of ADM to continue its extension until the upper pole, to cover the anatomical implant. In this study, we report the use of the technique described by Caputo et al. [9] on a patient. 

## 2. Case Report

**Figure 1:**
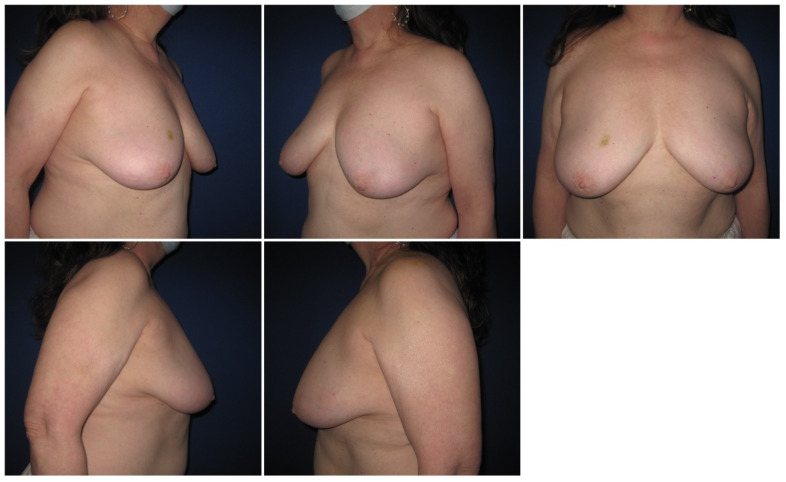
Pre-operative pictures.

The patient was a 62-year-old postmenopausal female with a history of indeterminate microcalcifications in the left breast (Figure 1). A biopsy confirmed intermediate grade, ER+/PR+/Her2— invasive ductal carcinoma. She denied breast pain, nipple discharge, overlying skin changes, or a mass on self-examination. She had taken hormone replacement therapy (HRT) for the previous 5 years. She discontinued her HRT after an abnormal mammogram. Her family history was significant for breast cancer, which was diagnosed in a paternal aunt. She denied any additional family history of breast or ovarian cancer. The patient was negative for a genetic mutation. Overall, she felt she was in good health for her age. Her BMI was 34.4, and she was a non-smoker and not hypertensive. She was doing all activities of daily living independently. The mammogram showed heterogeneously dense breast tissue, a grouping of pleomorphic microcalcifications in the upper outer left breast spanning 3.2 × 3.7 × 2.3 cm, associated architectural distortion, and a microcalcification tract anteriorly toward the nipple; scattered calcifications and subtle architectural distortion were also noted in the right breast. 

Bilateral whole breast ultrasound was performed. A 15 mm hypoechoic mass and 5 mm mass were present in the left breast at the 2 o’clock position 10 cm from the nipple corresponding with the pleomorphic microcalcifications. No suspicious sonographic findings were noted in the right breast. The axillary lymph nodes appeared normal bilaterally. 

She underwent breast MRI which was suggestive of multicentric disease in the left breast. 

Biopsy of the left breast demonstrated invasive ductal carcinoma, grade 2, with associated low-grade ductal carcinoma in situ, ER+ (80%) PR + (50%), and Her 2-, and patchy low-grade ductal carcinoma in situ. The final diagnosis was clinical stage IIA (T2 N0 M0), grade 2, ER+/PR+/HER2-, invasive ductal carcinoma of the upper outer quadrant of the left breast extending to the base of the left nipple. 

The patient desired single-stage reconstruction and elected for bilateral mastectomies with immediate pre-pectoral one-stage breast reconstruction with anterior coverage using superior anterior biological ADM and inferior dermal sling support. This type of reconstruction is not routinely offered, as the common reconstruction method is a 2-stage sub-pectoral breast reconstruction, which was an option in this patient, but the patient preferred one-stage breast reconstruction with pre-pectoral implants.

**Figure 2:**
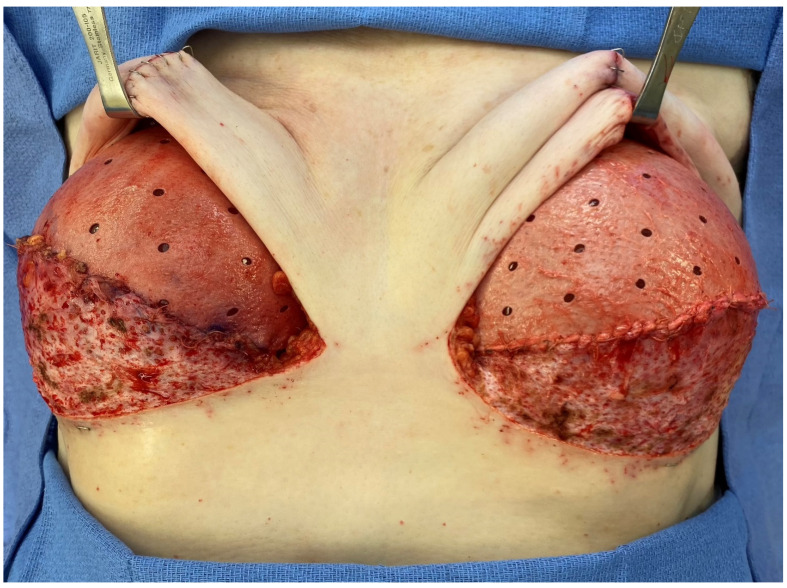
Intra-operative picture. For each breast, the superior-anterior part of the implant is covered by an ADM patch, sutured to a dermal sling that covers the inferior-anterior part of the implant.

She was considered a good candidate for total mastectomy, sentinel lymph node biopsy and immediate pre-pectoral one-stage breast reconstruction with anterior coverage using superior anterior biological ADM and inferior dermal sling support. This was discussed with the breast surgeon as well. She was not a good candidate for a nipple-sparing approach due to the likely involvement of the left nipple and pre-morbid ptosis. The patient underwent mastectomy and reconstruction using the technique described by Caputo et al. [9] (Figure 2 shows an intra-operative picture).

**Figure 3:**
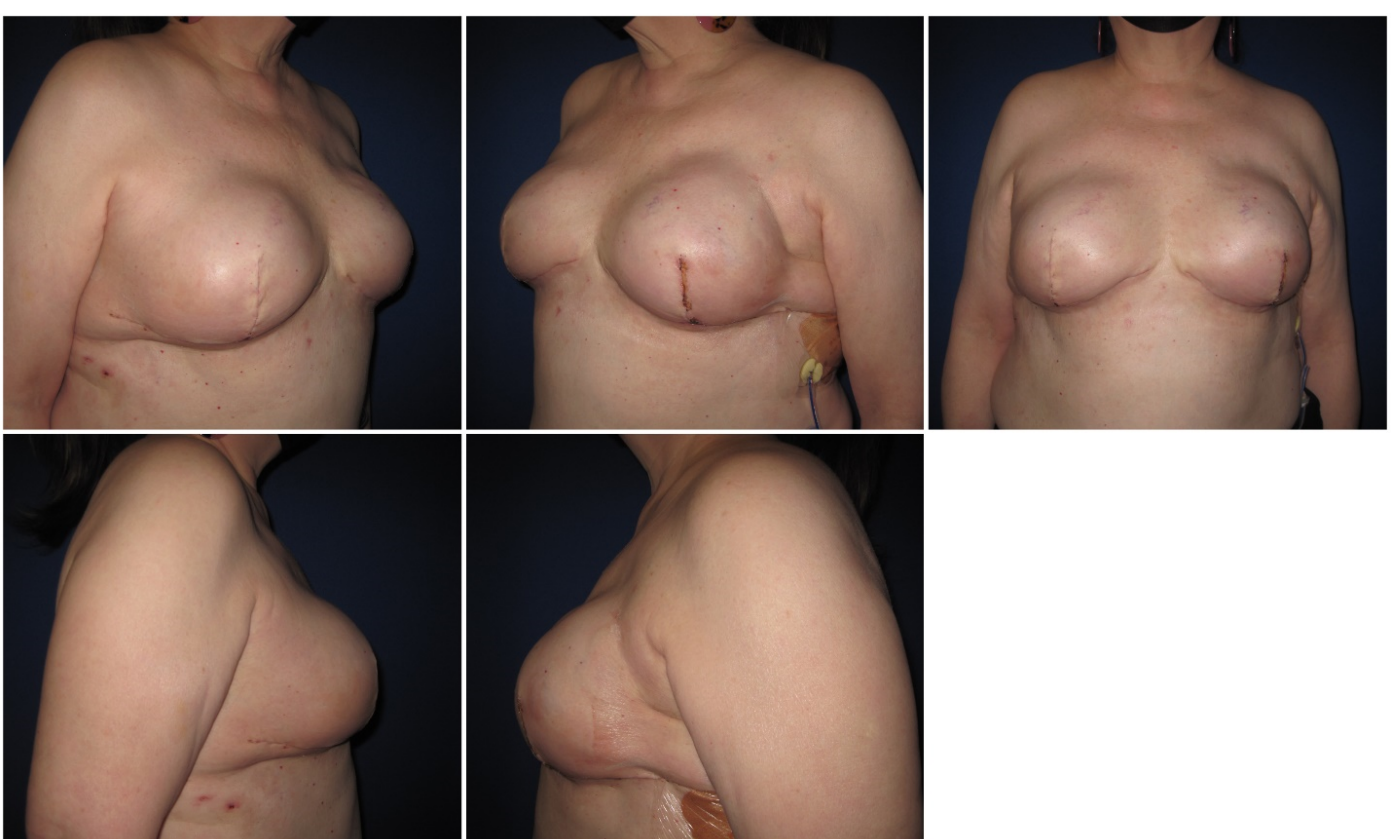
Immediate post-operative pictures.

**Figure 4:**
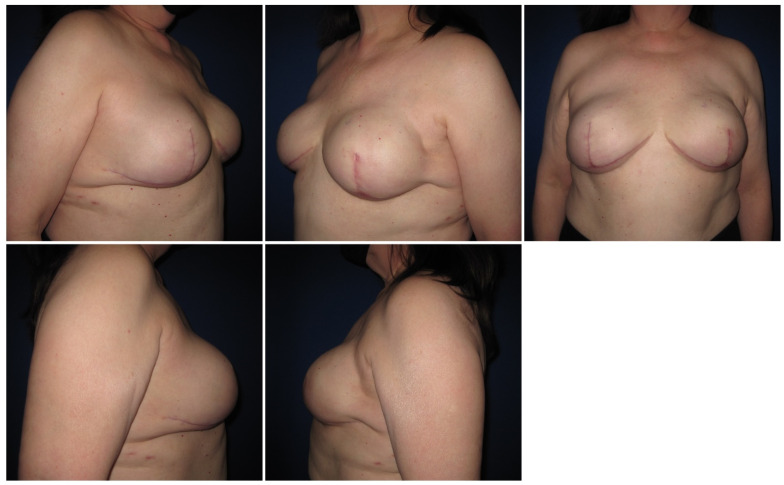
Post-operative pictures, 3 months after surgery.

Six months after surgery, she was satisfied, and she did not report any complications (Figure 3 shows immediate post-operative pictures and Figure 4 shows pictures at the 3 months follow-up encounter). The patient signed the informed consent for the publication of this case report along with the pictures. The Administrative Section of the University of Tennessee Health Science Center (UTHSC) Institutional Review Board (IRB) determined that this retrospective case study qualifies for NHSR (not human subjects research) status in that it does not involve “research” as defined in 45CFR46.102(l).

## 3. Surgical Technique

The patient underwent bilateral mastectomy with Wise pattern skin reduction, left axillary sentinel lymph node biopsy and implant and ADM pre-pectoral reconstruction with inferior dermal sling support. 

We used ADM to cover the superior-anterior part of the breast implant and a dermal sling to cover the inferior part of the implant (Figure 2) in a one-stage pre-pectoral breast reconstruction, as described by Caputo et al. [9]. 

The dermal flap of the lower pole was de-epithelialized and preserved. A combined dermal pocket was created using the inferior dermal flap, sutured with a patch of ADM to continue its extension until the upper pole, for total implant coverage.^9^

**Figure 5:**
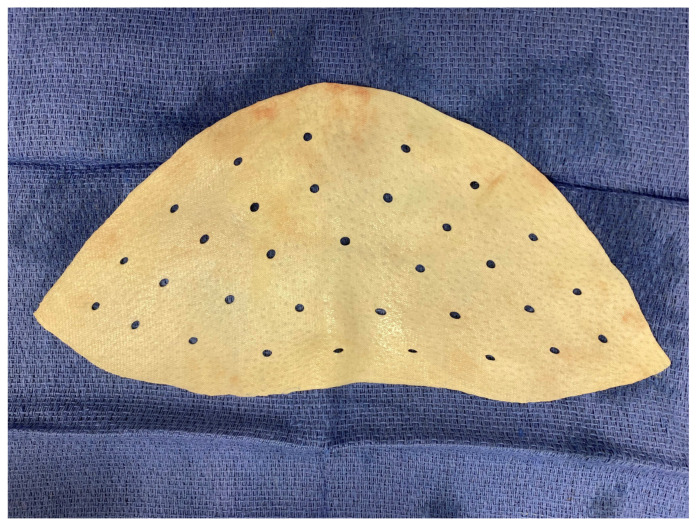
The ADM patch used (one for each breast) to cover the superior-anterior part of the implant. AlloDerm SELECT Regenerative Tissue Matrix, Contour Medium Perforated, thickness medium 1.6 mm ± 0.4 mm was used (ALLODERM and its design are trademarks of LifeCell Corporation, an AbbVie company).

AlloDerm SELECT Regenerative Tissue Matrix, Contour Medium Perforated, thickness medium 1.6 mm ± 0.4 mm was used (Figure 5).

## 4. Discussion

We presented a case of inverted T mastectomy with immediate one-stage breast reconstruction using ADM on the upper pole of the breast and dermal vascularized sling support for the inferior pole from the inframammary fold under the equator of the breast, suturing this one with the inferior margin of the ADM. 

This technique allows a breast pocket to be shaped in the subcutaneous space over the pectoralis major muscle in a Wise pattern inverted-T skin reduction mastectomy. The technique was originally described by Caputo et al. [9] in 2016. 

Later in 2019, Thuman et al. [10] reported the use of the same technique for pre-pectoral, two-stage breast reconstruction with Wise pattern skin reduction for patients who have a high BMI.

This technique offers single-stage immediate reconstruction, with a decreased requirement for ADM, increased use of vascularized tissue and implant support. Additionally, in the pre-pectoral space, decreased pain postoperatively and less anatomic disruption is offered. 

The concept of breast reconstruction has radically changed over the years, and currently, the possibility of obtaining an immediate breast reconstruction (IBR) is desirable in order to reduce the number of surgeries and the cost to the healthcare system. On the other hand, IBR with implants might cause skin stretching, possibly leading to skin flap necrosis, especially at the T-junction [11].

As shown by many authors, IBR has similar post-operative complications compared to delayed breast reconstruction [8]. The use of ADM to cover the breast implant led to a new perspective both in submuscular and in pre-pectoral IBR [8]. In the submuscular IBR, the use of ADM allows for better lateral control of breast implant without serratus muscle mobilization and/or lower coverage in the dual-plane reconstruction with long-term cosmetic results [8].

Before the introduction of ADMs, the muscular coverage of the implant was a must. In the pre-pectoral IBR, ADM is the keystone to spare pectoralis major use and, consequently, to reduce the risk of upper migration of the implant and pain due to muscle detachment [8,12]. Pre-pectoral breast reconstruction is generally suggested in those patients where implants are less than 500 ccs, although some authors have described pre-breast reconstruction with implants over 600 cc [8]. Many surgeons choose the pre-pectoral breast reconstruction because the submuscular placement of the implant can lead to a “contrived breast”. This aspect is relevant and linked to a loss of muscle function. In 2007, the use of ADM in pre-pectoral implant-based reconstruction was firstly described by Bindingnavele et al. [13].

ADM is a biotechnologically designed human tissue of bovine or porcine origin in which tissue processing removes cellular antigens. This process diminishes an immunological response while maintaining a structural matrix that promotes angiogenesis and tissue regeneration [14]. Advantages of ADM use in breast reconstructive strategies include tissue support, reducing the inflammatory response, reducing the probability of capsular contracture occurrence, and reducing the risk of malposition. It has been demonstrated that the levels of myofibroblasts are significantly lower in ADM capsules than in submuscular capsules [15]. In particular, structural support and pocket definition have been hypothesized to help decrease rates of capsular contracture by ADM utilization. The downside of ADM use is the high cost and potential risk of infection, even though different reports and opinions have been published regarding this matter [8].

**Figure 6:**
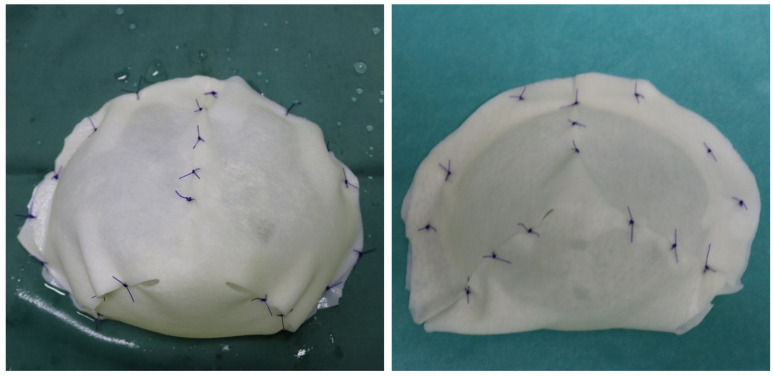
Pictures showing an example of total implant wrapping using ADM (acellular dermal matrix) from Dr. Cuomo’s archive.

The feasibility of a true muscle-sparing procedure with subcutaneous placement of a completely ADM-wrapped implant (Figure 6) is common and has been investigated by several authors, reporting high aesthetic outcomes and patients’ satisfaction [8,16,17].

A Wise pattern mastectomy with a de-epithelialized dermal sling and submuscular direct-to-implant reconstruction to optimize implant-based reconstruction in patients with large ptotic breasts has been described by several authors [9,18]. The combination of a skin-reducing mastectomy through a Wise pattern incision and ADM implant reconstruction has been recently investigated in patients with large ptotic breasts [9]. A pre-pectoral pocket with a dermal flap overlapped to ADM for lower- and upper-pole coverage of the breast implant has been described by Maruccia et al. (the inferior dermal flap is sutured on the anterior surface of the ADM) [19]. The combination of an acellular dermal matrix and a dermal sling has been investigated by KanKam et al. [18], providing a double-layer ‘water-proofing’ and support for the implants inferiorly in order to avoid the T-junction breakdown complication.

The technique described in our case report is focused on the use of inverted-T Wise pattern mastectomy, which allows obtaining good aesthetic outcomes in patients with large and ptotic breasts [9,10]. Breast ptosis, in fact, is a very frequent event in women undergoing mastectomy, as breast cancer usually arises from the age of 40 years [1]. The use of the inverted-T technique allows for restoring the harmony of the mammary region by reshaping the projection of the breast and the re-position of the areola-nipple complex after mastectomy in cases of the nipple-skin sparing technique. 

However, this type of mastectomy is linked to a higher risk of necrosis of the T joint and, consequently, risk of exposure of the deep tissues. 

The limitation of this study is that this is a case report on one single patient. Future research is needed to add data to the ones available from this case report and from the previous studies that described the use of this surgical technique [9,10] for breast reconstruction.

## 5. Conclusions

Taking all of these advancements in techniques, we were able to offer our patient a single-stage reconstruction with improved shape via the Wise pattern skin reduction in a pre-morbid ptotic breast. The pre-pectoral space offered less disruption of her anatomy, sparing the pectoralis major and serratus anterior muscles with improved comfort in the post-operative period. The dermal support flap was two-fold, offering mechanical support to the weight of the implant as well as vascular support to the overlying skin of the Wise pattern T-junction. Additionally, the use of the autologous tissue inferiorly decreased the amount of ADM required, which lowered the cost as well. This technique can be considered to improve outcome and reconstructive option offerings to a larger, ptotic breast as a safe and cosmetically pleasing alternative.

## Data Availability

The datasets used in this current study are available from the cor-responding author on reasonable request.
